# Evaluating upper limb impairments in multiple sclerosis by exposure to different mechanical environments

**DOI:** 10.1038/s41598-018-20343-y

**Published:** 2018-02-01

**Authors:** Laura Pellegrino, Martina Coscia, Margit Muller, Claudio Solaro, Maura Casadio

**Affiliations:** 1 Department Informatics, Bioengineering, Robotics and Systems Engineering, University of Genoa, Genoa, Italy; 2 Bertarelli Foundation Chair in Translational Neuroengineering, Ecole Polytechnique Federale de Lausanne, Lausanne, Switzerland; 3 Wyss Center for Bio- and Neuroengineering, Geneva, Switzerland; 4 Azienda Sanitaria Locale di Genova, ASL3 Genoa, Italy

## Abstract

Multiple sclerosis is a chronic, autoimmune and neurodegenerative disease affecting multiple functional systems and resulting in motor impairments associated with muscle weakness and lack of movement coordination. We quantified upper limb motor deficits with a robot-based assessment including behavioral and muscle synergy analysis in 11 multiple sclerosis subjects with mild to moderate upper limb impairment (9 female; 50 ± 10 years) compared to 11 age- and gender- matched controls (9 female; 50 ± 9 years). All subjects performed planar reaching tasks by moving their upper limb or applying force while grasping the handle of a robotic manipulandum that generated four different environments: free space, assistive or resistive forces, and rigid constraint. We recorded the activity of 15 upper body muscles. Multiple sclerosis subjects generated irregular trajectories. While activities in isolated arm muscles appeared generally normal, shoulder muscle coordination with arm motions was impaired and there was a marked co-activation of the biceps and triceps in extension movements. Systematic differences in timing and organization of muscle synergies have also been observed. This study supports the definition of new biomarkers and rehabilitative treatments for improving upper limb motor coordination in multiple sclerosis.

## Introduction

Multiple sclerosis (MS) is a chronic and autoimmune disease, affecting the white and gray matter in the central nervous system (CNS)^1^. MS typically affects multiple functional systems, thus resulting in a variety of symptoms that significantly impact the quality of life, including sensory deficits, muscle weakness and lack of movement coordination^2–4^.

Sensorimotor impairments of the lower limbs affect the mobility in 75% of MS cases^5^. Since these are often the first impairments to occur, most studies on MS focus on the lower limbs^6–8^. However, 66% of the MS population also have upper limb motor impairments^9^ that dramatically affect many daily living activities^10^. An accurate assessment of upper limb movements is challenging due to their inherent variability. Complex technological solutions are often needed to define controlled environments for capturing a broad range of arm motion strategies.

Technology-aided neurorehabilitation has evolved greatly over the past few decades and we are now in a position to improve the understanding of motor recovery and to provide treatment after neurological injuries^11^. Robotic systems are used for motor training, but they also represent excellent platforms to control the task, providing different external forces and allowing quantitative and repeatable motor performance measures that can be used to assess motor recovery^12–18^. Some studies have analyzed the kinematic performance during planar reaching movement in MS subjects by means of a graphic tablet^19^ or a planar robotic manipulandum^12,15,18,20^. Two studies have reported the usefulness of end-effector robots as assessment tools for quantifying motor coordination in symptomatic MS subjects during reaching tasks towards virtual targets on a screen^12,16^.

The evaluation of behavioral parameters together with the measure of neurophysiological signals, such as the electromyographic activity, opens the possibility for a comprehensive characterization of the origin, the expected prognosis and the functional consequences of motor impairments after a specific neurological injury.

Recently, muscle synergies have been used to describe muscle coordination features related to motor impairments in neurological diseases^21,22^. Muscle synergies describe how groups of muscles are concurrently activated to perform a motor task. Different factorization algorithms^23,24^ are used to extract them from surface electromyography signals (EMG). In healthy individuals, muscle synergies explain a large number of spatiotemporal characteristics of muscle patterns recorded in upper limb during center-out reaching movements^25–27^. Moreover, muscle synergies can be a useful tool for clinical applications^28,29^. In the affected side of severe chronic stroke survivors, upper limb muscles synergies can be modified and their level of preservation correlates with the level of motor impairment^30,31^. Remarkably, in sub-acute stroke survivors after treatment consisting in robot-assisted planar reaching movements, the altered muscle synergies were modified and they resembled ones of healthy individuals^32^.

Therefore, we hypothesize that muscle synergies analysis may be used together with behavioral parameters to quantify upper limb motor impairments in MS. To the best of our knowledge, muscle synergies have not previously been investigated during upper limb tasks in the MS population, although there is some evidence that for lower limbs the muscle activations and the synergies are systematically modified in relation to walking impairment, muscle weakness, and prolonged double support^33–35^.

Here, we propose a method to evaluate behavioral parameters, muscle activity and coordination, using muscle synergy analysis, of both arms in MS and healthy subjects while they control their hand motion and/or forces in a task with different mechanical environments. We used a planar robotic manipulandum for a quantitative and repeatable assessment of upper limb motion and force strategies, as well as a means to test which task features might influence the assessment parameters. We included different mechanical environments and movement directions in order to maximize the variability of reaching movements and to generalize the results. We separately considered dominant and non-dominant arm because during the disease, approximately three out of four MS subjects are affected by bilateral upper limb dysfunction^5^. In this way, we aim to identify behavioral and muscular features that are sensitive to extrinsic constraints (i.e. different environments), to intrinsic motor control constraints (i.e. dominant vs. non-dominant side) and, most importantly, to the disease.

## Results

### Overview

In this study we investigated performance parameters, muscle activity and coordination in eleven MS subjects (MS: 9 Female; 50 ± 10 years, Table 1) and eleven age and sex matched controls (C: 9 Female; 50 ± 9 years) carrying out upper limb reaching tasks in four different mechanical environments.

**Table 1:** Multiple sclerosis subjects' features.

	**Age**	**Sex**	**EDSS (0–10)**	**9-HPT (s)**		**FSS (0–63)**
				Right	Left	
**S1**	40	M	6.5	45.96	85.15	59
**S2**	42	F	6.5	94	76	42
**S3**	44	M	6.5	73.4	75.6	33
**S4**	55	F	2.5	21	24.5	45
**S5**	61	F	5	23	27.65	53
**S6**	64	F	6	43.85	45.6	51
**S7**	46	F	5	23.94	50.23	58
**S8**	67	F	6.5	41.85	94	51
**S9**	47	F	5	24	28	45
**S10**	39	F	6.5	28	37	61
**S11**	42	F	5	40.21	50.29	22

EDSS is for Expanded Disability Status Scale; 9-HPT for the Nine Hole Peg Test; and FSS for the Fatigue Severity Scale. M = Male, F = Female.

**Figure 1:**
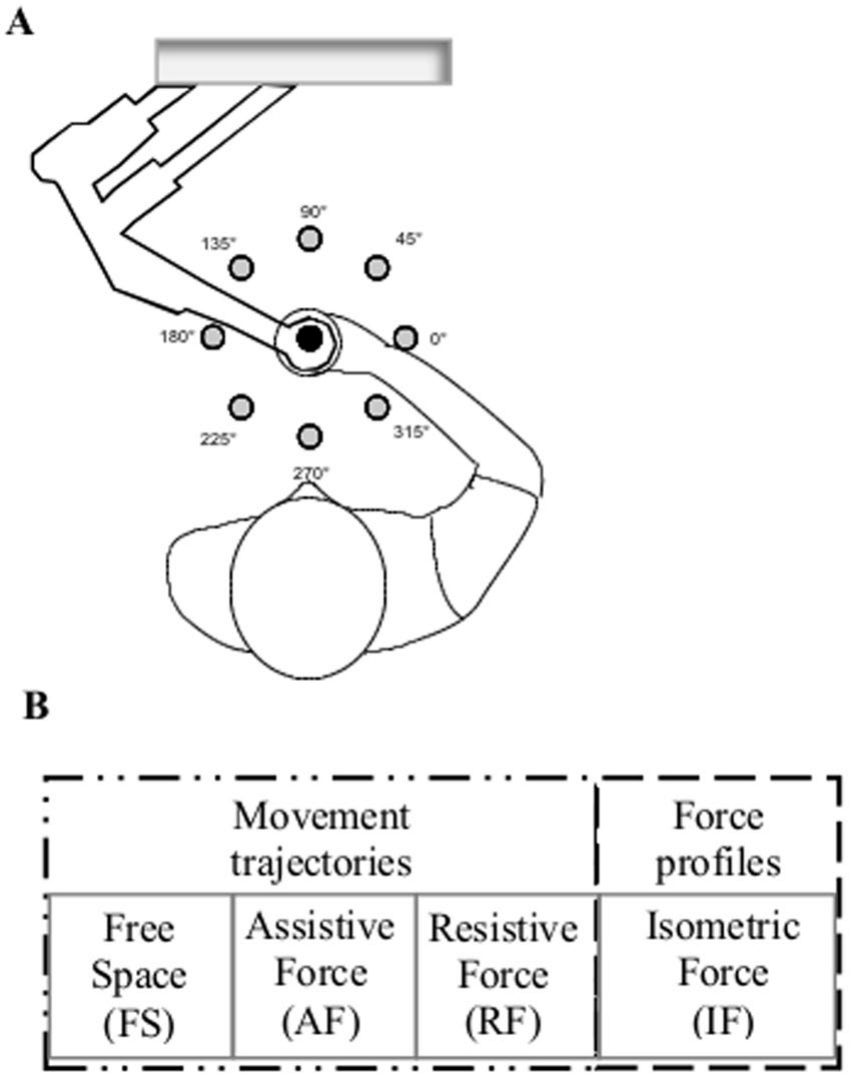
Experimental set-up and protocol. (**A**) Schematic representation of the experimental setup and targets positions. The circles represent the positions of the peripheral targets on the computer screen; in black is the home target, in grey the eight targets presented during the experimental protocol. (**B**) Experimental protocol. Tasks in different mechanical environments (from left to right): free space (FS), assistive force (AF), resistive force (RF) and isometric force (IF).

Subjects controlled a cursor on a computer screen by moving their hand or applying force. They had to reach, by center-out movements, targets that appeared in eight directions equally spaced and at a fixed distance from a central target (Fig. 1A). Specifically, subjects performed three motion tasks moving their hand in presence of 1) no external forces (free space, FS), 2) an assistive force (AF), and 3) a resistive force (RF). In the fourth task, they exerted an isometric force (IF) against a rigid constraint (Fig. 1B). Subjects were asked to reach the targets as accurately as possible, without time constraints, thus they performed the task at their self-selected speed. Subjects performed the tasks with both the dominant (D) and the non-dominant (ND) arm.

The activities of the following 15 muscles were recorded for each upper limb: triceps brachii long (TRLO) and lateral head (TRLA), biceps brachii short (BICS) and long head (BICL), brachioradialis (BRAD), brachialis (BRA), pronator teres (PRON), infraspinatus (INFR), latissimus dorsi (LATI), upper trapezius (TRAP), rhomboid major (RHOM), pectoralis major (PECT), anterior (DANT), medial (DMED) and posterior (DPOS) deltoid.

### Behavioral Analysis

**Figure 2:**
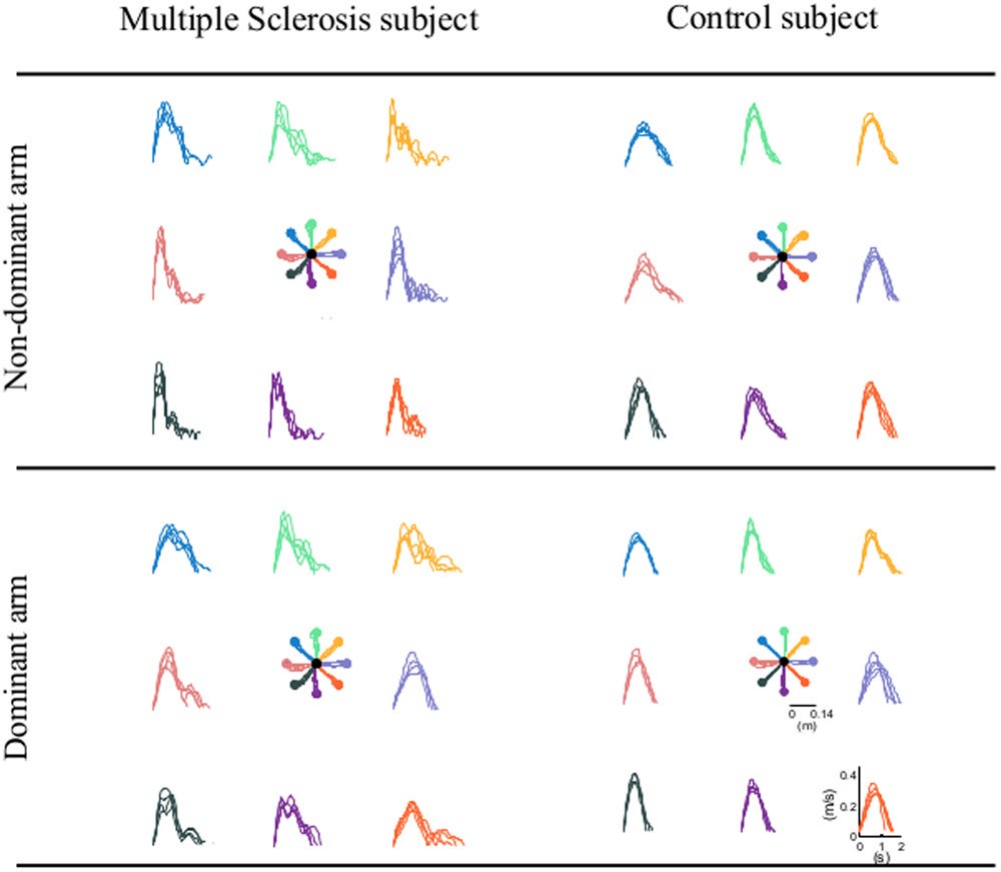
Movement trajectories and speed profiles in the free space task for a multiple sclerosis subject (first column) and matched control subject (second column). Top row: non-dominant arm. Bottom row: dominant arm.

As expected, when compared to controls, the MS subjects had worse performance. All MS subjects had cursor trajectories characterized by more corrections in the second part of the movements, i.e. during the deceleration phase for the movement tasks (Fig. 2).

**Figure 3:**
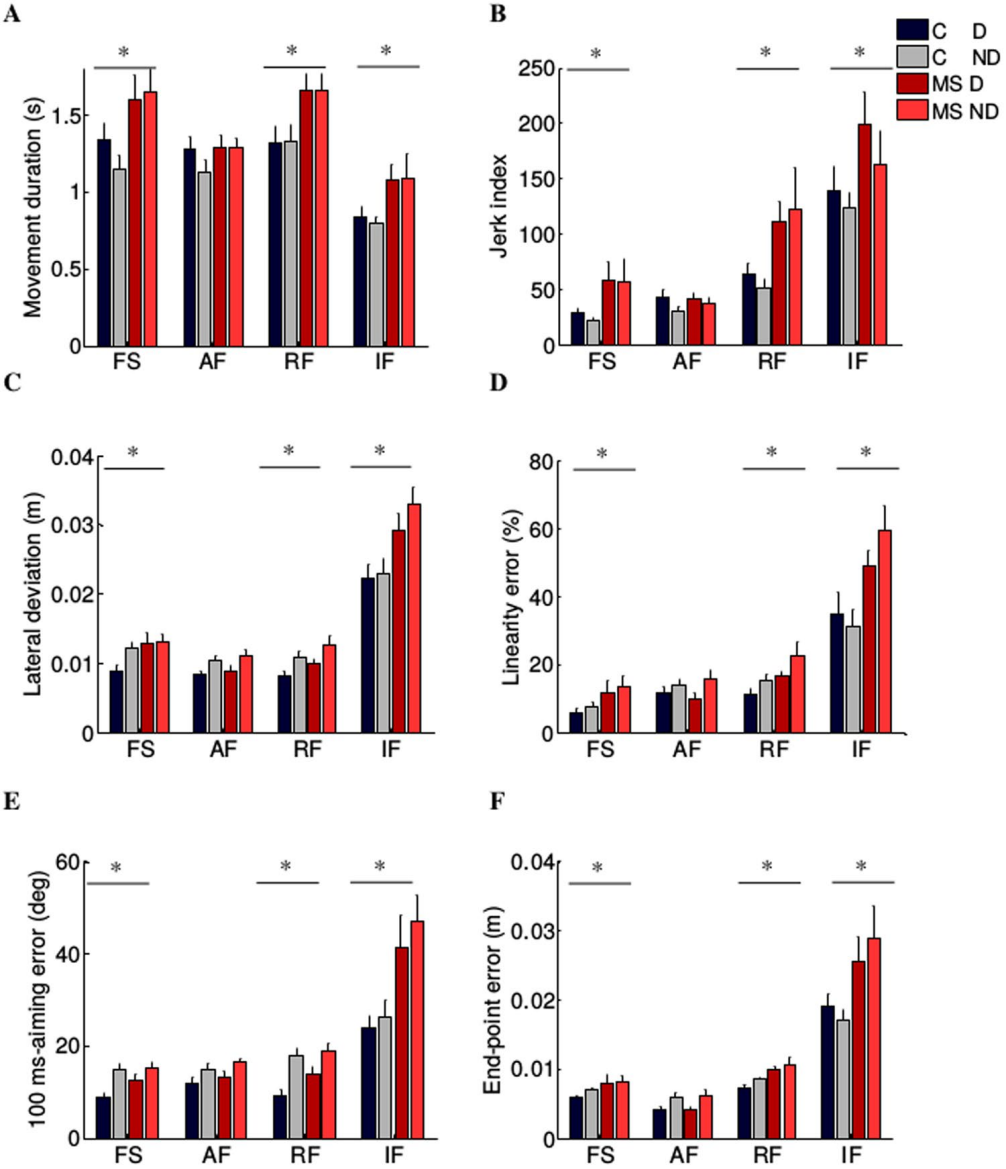
Behavioral indicators of the cursor trajectories during the motion tasks (motion trajectories) in absence of external force (FS), in presence of assistive (AF) or resistive (RF) force and during the isometric task (IF, force profiles). Control subjects (C) and MS subjects (MS) are shown with different colors as indicated in the legend. Darker and brighter colors represent the dominant (D) and non-dominant (ND) arm respectively. The error bars indicate the standard error of the indicators. * indicates significant differences (p < 0.05) between subject groups (C vs MS) for each task.

The duration of each reaching movement for the MS subjects was significantly higher (F(1,20) = 6.33, p = 0.02; Fig. 3A), the average speed lower (F(1,20) = 5.26, p = 0.04) and the trajectories were less smooth (jerk index: F(1,20) = 7.58, p = 0.01; Fig. 3B). MS subjects were also less accurate: their trajectories had larger lateral deviations (F(1,20) = 17.10, p = 0.01; Fig. 3C) and greater linearity error (F(1,20) = 9.86, p = 0.005; Fig. 3D). The errors were relevant both at the beginning (100-ms aiming error: F(1,20) = 8.41, p = 0.001; Fig. 3E) and at the end of the trajectory (end-point error: F(1,20) = 15.76, p = 0.01; Fig. 3F).

The difference in performance between the populations depended on the task (disease $\times$ task effect - movement duration: F(3,60) = 5.43, p = 0.04, average speed: F(3,60) = 11.80, p = 0.03; jerk index: F(3,60) = 10.15, p = 0.03; lateral deviation: F(3,60) = 5.41, p = 0.002; end-point error: F(3,60) = 13.63, p = 0.01; linearity error: F(3,60) = 5.04, p = 0.004; 100 aiming-error: F(3,60) = 6.41, p = 0.001).

With the assistive force, the two populations behaved similarly (i.e. no significant differences were found comparing the performance parameters of the two groups; post-hoc - movement duration: p = 0.47; average speed: p = 0.15; jerk index: p = 0.94; lateral deviation: p = 0.41; linearity index: p = 0.99; 100-ms aiming error: p = 0.43 and end-point error: p = 0.57). Instead, in the other tasks the movements of the MS subjects had longer duration (post-hoc - FS: p = 0.01, RF: p = 0.01 and IF: p < 0.001), were slower (average speed - FS: p = 0.02, RF: p = 0.01 and IF: p = 0.45), were less smooth (jerk index - FS: p = 0.002, RF: p = 0.003 and IF: p < 0.001) and less accurate in terms of lateral deviation (FS: p = 0.03, RF: p = 0.02 and IF: p = 0.003), linearity error (FS: p = 0.002, RF: p = 0.002 and IF: p < 0.001), 100ms-aiming (FS and RF: p = 0.001 and IF: p < 0.001) and end-point error (FS: p = 0.011, RF: p = 0.002 and IF: p < 0.001). Both populations had more difficulty controlling a force trajectory than a movement trajectory, i.e. they had worse performance during the IF task than during the movement tasks. Moreover, the differences between the two populations in terms of accuracy were greater when subjects controlled isometric force compared to movement execution (Fig. 3).

We did not find any difference in the behavioral indicators between the two populations with respect to the D and ND arm (disease $\times$ arm effect). However, this result was biased by the fact that the MS subjects could have higher impairment either in the dominant or in the non-dominant side. To account for this, we computed the absolute difference between the performance indicators of the two arms for each subject and then compared the two groups. We found a significant difference between the two populations. Specifically, MS subjects had less symmetric performance between the two arms in terms of duration, jerk index and final error in all tasks, except when in presence of the assistive force (disease effect- movement duration: F(1,20) = 6.16, p = 0.03; jerk index: F(1,20) = 14.23, p = 0.04; end-point error: F(1,20) = 4.15, p = 0.04, Fig. S1A,B and S1F), and in all the movement tasks for the other accuracy indicators (lateral deviation: F(1,20) = 5.05, p = 0.04; linearity index: F(1,20) = 5.77, p = 0.02; 100-ms aiming error: F(1,20) = 7.46, p = 0.03; Fig. S1C and E).

### EMG activation

Muscle activity in terms of amplitude modulation in the different tasks and directions as well as in terms of timing was similar in most muscles between MS and control groups.

However, the RHOM (for more details see Supplementary Fig. S2A) and DANT of the MS subjects had different amplitude modulation (disease effect: F(1,20) = 10.40, p < 0.001 and F(1,20) = 20.24, p < 0.001, respectively) and activation timing (r_EMG-ARM_ < 0.30 for both muscles in each task and arm) with respect to control subjects. In particular, MS subjects increased their activity while moving their hand toward the body, flexing their elbow, compared to distal directions that required the extension of the elbow (i.e. 225°, 270° and 370°; disease $\times$ direction effect: F(7,140) = 12.38, p < 0.001). These differences were observed in both arms during the movement tasks, and increased in the RF task. Moreover, most MS subjects had an abnormal activation of the BICL (disease effect: RMS: F(1,20) = 21.20, p < 0.001, disease $\times$ direction effect: F(7,140) = 14.20, p < 0.001; and r_EMG-TASK_ < 0.40 in each arm) when reaching distal targets in the movement tasks, i.e. MS subjects co-activated the antagonist muscles BICL and TRLO during movements that required the elbow extension (for more details see Supplementary Fig. S2B,C).

### Muscle synergies

We used the non-negative matrix factorization (NNMF) algorithm to extract muscle synergies from the EMG envelopes^24,30,31,36–39^. In this view, a muscle synergy represents a set of muscles, which are simultaneously activated by a single temporal command. The organization of a synergy is determined by the contribution (i.e. weight coefficient) of each muscle, as specified by the weight matrix W. Its activation profile is defined by the activation coefficients, specified by the matrix H.

The number of synergies was not significantly different between populations (F(1,19) = 1.23, p = 0.48) and between D and ND arms for both populations (F(1,20) = 1.77, p = 0.38).

In the movement task (i.e. FS, AF and RF), five muscle synergies were extracted for D (FS: 5.4 ± 0.5SE, AF: 5.2 ± 0.3SE and RF: 4.9 ± 0.4SE) and ND arm (FS: 5.1 ± 0.4SE, AF: 4.9 ± 0.4SE and RF: 4.9 ± 0.3SE) of control subjects. Similarly, five muscle synergies were identified for D (FS: 4.9 ± 0.4SE, AF: 5.1 ± 0.2SE and RF: 5.1 ± 0.6SE) and ND arm (FS: 5.1 ± 0.3SE, AF: 4.9 ± 0.3SE and RF: 5.1 ± 0.4SE) of the MS subjects.

**Figure 4:**
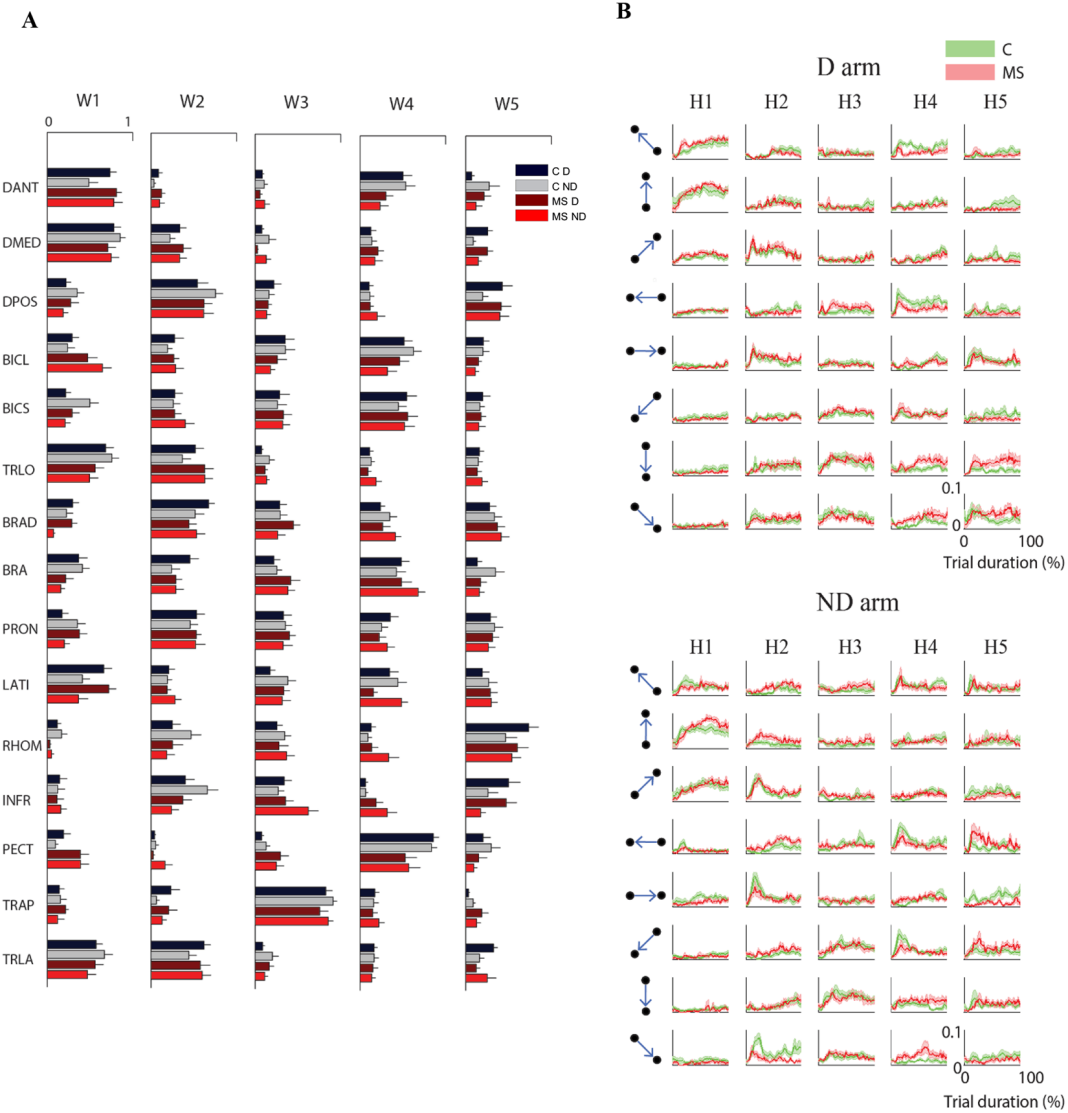
Weight and activation coefficients of the muscle synergies during the task in free space without external forces. (**A**) Weight coefficients for all muscle synergies (W1 to W5). Weight coefficients are shown for the two arms (dark: Dominant (D) and light: non-dominant (ND)). Control subjects (C) and MS subjects (MS) are shown with different colors as indicated in the legend. The error bars represent the standard error. (**B**) The green and red line represents the mean activation profiles for control subjects and MS subjects, respectively, for the five synergies (H1 to H5) in the dominant (D, first panel) and non-dominant (ND, second panel) arm. The shaded area indicates the standard error.

The organization of these five muscle synergies had the following characteristics in both populations (Fig. 4A,B):

- Synergy 1 involved the DANT, DMED, TRLO and TRLA. This synergy was mainly active during movements directed toward 45°, 90° and 135°. This synergy contributed to the abduction and flexion of the shoulder and the extension of the elbow.
- Synergy 2 involved the DPOS and PRON. This synergy was mainly active during movements directed toward 0° and 45°. This synergy contributed to the extension of the shoulder.
- Synergy 3 was a muscle-specific synergy dominated by the activity of the TRAP, with minor contributions from the other muscles. This synergy facilitated the stabilization of the shoulder.
- Synergy 4 included the BICL, BICS and PECT. This synergy was mainly active during movements toward 225°, 270° and 315°. This synergy contributed to the flexion of the elbow.
- Synergy 5 included the RHOM and INFR. This synergy facilitated the stabilization of the shoulder.

In contrast, there was a significant difference in the number of muscle synergies for both groups between the isometric task and the movement tasks (F(3,60) = 12.45, p < 0.001; post-hoc: FS vs IF, AF vs IF and RF vs IF: p < 0.001). In the isometric task (i.e. IF), only four muscle synergies were identified for controls (D: 4.1 ± 0.3SE and ND: 4.3 ± 0.3SE) and MS subjects (D: 4.2 ± 0.4SE and ND: 4.0 ± 0.4SE). Specifically, the muscle-specific synergy (Synergy 3 in the movement tasks) was absent. In IF, synergy 2 included the muscles responsible for the stabilization of the shoulder (TRAP, RHOM and INFR) and for the posterior-position of the shoulder (DPOS and DMED).

#### Organization of the muscle synergies (weights, W)

Both populations changed the organization of the muscle synergies in response to the mechanical environment that characterized each task. Specifically, MS subjects during movement tasks changed to a greater extent the organization of their muscle synergies in the presence of external forces.

**Figure 5:**
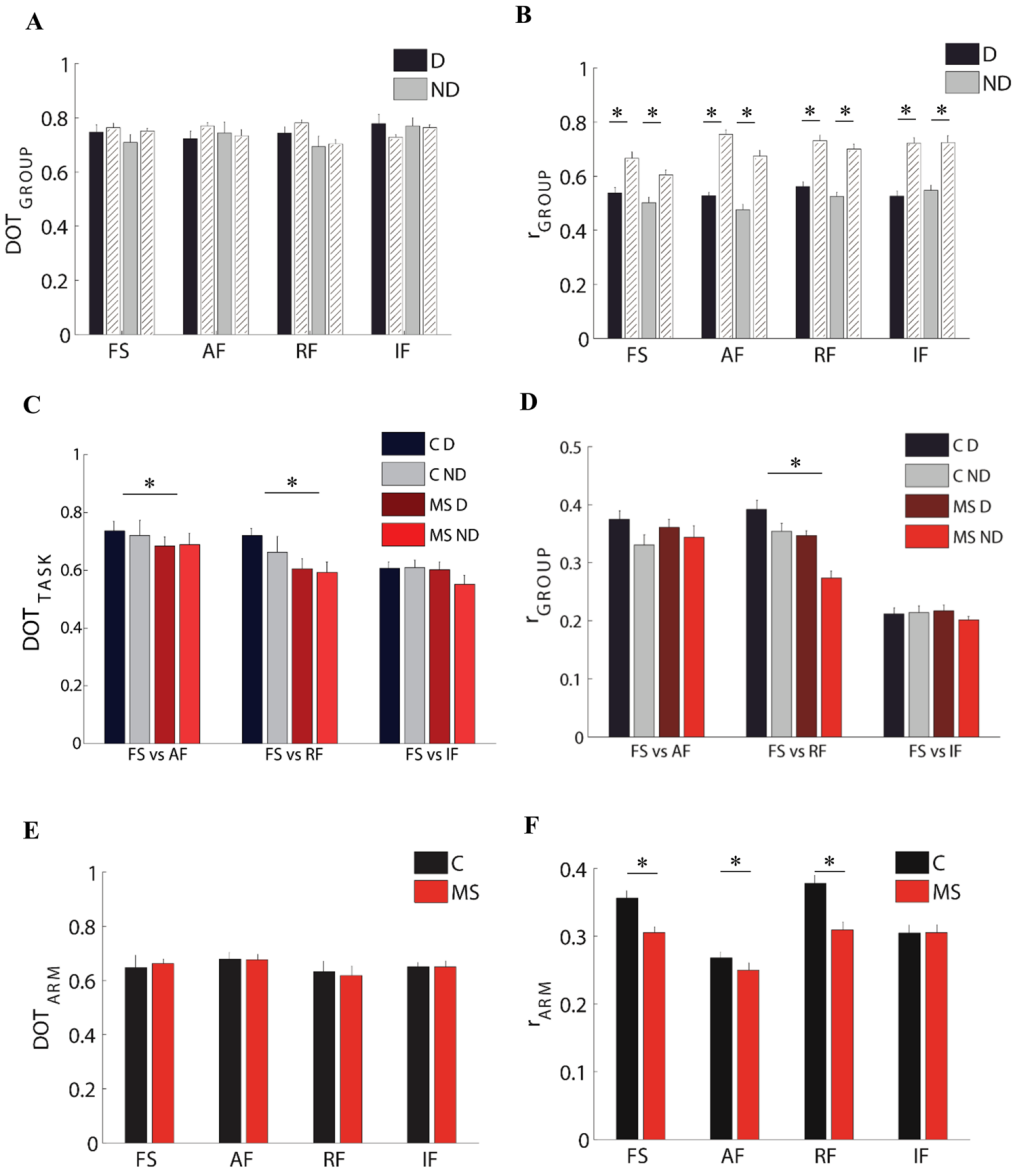
Comparison of weight and activation coefficients of the muscle synergies between groups, tasks, and arms. Left columns: comparison of weight coefficients by the scalar product (DOT). Left columns: comparison of activation coefficients by the Pearson correlation (r). First row: MS subjects compared to control subjects i.e. inter-groups indicator for the dominant (D, black bars) and the non-dominant (ND, gray bars) arm compared to the same indicator computed intra-group (white bars). Second row: comparison of the FS task with the AF, RF and IF tasks respectively, both for the dominant (D) and non-dominant (ND) arm. Third row: comparison between the two arms for MS subjects (MS, red bars) and control subjects (C, black bars). The error bars represent the standard errors. * indicates significant differences (p < 0.05) between subject groups.

This result was highlighted by the DOT metrics (i.e. the scalar product between pairs of weight synergy vectors^26,36^; see materials and methods) computed both for comparing the subject groups, DOT_GROUPS,_ and for comparing the group performance in the different tasks, DOT_TASK_. As for the former_,_ while we did not find a significant absolute difference between the DOT_INTER-GROUPS_ -obtained with comparisons between the two groups- and the DOT_INTRA-GROUP_ -obtained with comparisons within the control group (F(1,10) = 1.36, p = 0.26; Fig. 5A), we found a significant interaction effect between these two parameters and the task (F(3,30) = 7.69, p = 0.04).

As for the DOT_TASK_ metric, when comparing the organization of the muscle synergies across tasks, we found a significant difference between populations (F(1,19) = 13.06, p = 0.04; Fig. 5C). As predicted by the difference in behavior, we found a low level of similarity when comparing FS with IF for both groups (task effect: F(2,40) = 12.51, p < 0.001). However, in the control subjects the comparison between reaching movements in free space and isometric force control resulted in a greater difference (FS vs IF - D: 0.60 ± 0.02SE and ND: 0.61 ± 0.02SE) than the comparison between reaching movements in the absence or presence of either assistive forces (FS vs AF: D: 0.73 ± 0.03SE and ND: 0.72 ± 0.05SE) or repulsive forces (FS vs RF: D: 0.71 ± 0.02SE and ND: 0.66 ± 0.04SE). In contrast, the MS subjects presented a higher change in the organization of muscle synergies (disease $\times$ task effect: F(1,40) = 12.30, p = 0.01), not only comparing the free space and isometric tasks (FS vs IF: D: 0.60 ± 0.02SE and ND: 0.55 ± 0.03SE), but also when they performed the same reaching movements in presence of different forces (FS vs AF- D: 0.68 ± 0.03 SE, ND: 0.68 ± 0.04SE and FS vs RF- D: 0.60 ± 0.03SE, ND: 0.59 ± 0.03SE).

In the comparison of D and ND arm (DOT_ARM_), we did not find any difference in weight coefficients between controls and MS subjects in all tasks (disease effect: F(1,19) = 1.15, p = 0.29; Fig. 5E).

#### Activation profiles of muscle synergies (H)

Finally, we compared by Pearson correlation^32,40^ (see materials and methods) the activation profiles of muscle synergies (Fig. 4B) between the two populations (r_GROUP_), among tasks (r_TASK_) and between D and ND arm (r_ARM_).

As for the comparison between the two populations, we found a significant difference in the activation profiles of muscle synergies between the r_INTER-GROUPS_ and r_INTRA-GROUP_ (F(1,10) = 52.19, p < 0.001; Fig. 5B) and this difference was also task-dependent (interaction effect: F(3,30) = 31.15; p < 0.001).

As for the comparison among tasks, the correlation between the activation profiles of two different task, r_TASK_, was significantly different between control and MS groups (F(1,10) = 10.16, p = 0.04) and this difference depended also on the pair of tasks that we compared (F(2,40) = 11.09, p = 0.03). As expected, we specifically observed a lower level of similarity when comparing the activation profiles in the FS vs IF tasks than when comparing FS vs AF or FS vs RF for both populations (task effect: F(2,40) = 18.07, p < 0.001; Fig. 5D). Consistent with what was observed for the organization of muscle synergies, the MS subjects also changed the activation profiles to a greater extent than the control groups, when comparing movements with and without repulsive forces (post-hoc: p = 0.001).

As for the comparison between D and ND sides, the correlation between the activation profiles of the two arms (r_ARM_) for both control and MS groups was task-dependent (task effect: F(1,20) = 4.74, p = 0.01; Fig. 5F). For the controls, in the movement tasks (i.e. FS, AF and RF), r_ARM_ ranged from 0.37 ± 0.01SE in RF task to 0.35 ± 0.02SE for FS, and 0.26 ± 0.01SE in AF task. This result suggests that in the presence of resistive force, the activation profiles of muscle synergies were more similar between the two arms than in the presence of an assistive force or in free space. The D and ND arm of the MS subjects had the same trend (RF: 0.31 ± 0.01SE, FS: 0.30 ± 0.01SE, AF: 0.25 ± 0.01SE), but the correlation between D and ND arm in all movement tasks were significantly lower than in the controls (task $\times$ disease effect: F(3,30) = 3.56, p = 0.01; Fig. 5F). In the isometric task, the r_ARM_ was similar between controls and MS subjects (0.30 ± 0.02SE and 0.30 ± 0.03SE, respectively).

## Discussion

This study systematically investigates the differences in movement execution, muscle activity and coordination between MS subjects and their age and gender matched controls during upper limb reaching tasks in different mechanical environments. We identified behavioral and muscular features specifically sensitive to the MS disease. Moreover, we investigated if these features depended on the interaction with specific environments. Finally, we studied both the dominant and non-dominant side of the upper body for both populations to detect asymmetries due to limb dominance and to separate them from the asymmetries due to different impairments affecting the two arms.

MS subjects performed less effectively than controls. In accordance with previous studies^15,41^, hand movements and force trajectories of MS subjects were slower, less smooth and less accurate. Comparing lateral and linearity errors, MS subjects had greater errors both in the initial (100-ms aiming error) and in the final (end-point error) part of the force trajectory. Differences between populations in terms of accuracy in the movement tasks were less marked than reported in other studies^15,41,42^. This may be due to a difference in task demands: unlike previous studies, where subjects were asked to maintain an approximately constant velocity, we adopted self-selected speed and asked subjects to perform accurate movements. Thus, in the present study, MS subjects compensated for the greater level of difficulty to accomplish the task with longer execution time, resulting in slightly better accuracy.

The differences between the two populations were greater when subjects controlled isometric force compared to movement execution. Therefore, the isometric force task was more sensitive to differences caused by the disease and may thus be a better tool to differentiate between stages of the disease. Despite this, the study of force control has received less attention than the study of movement especially in people with neurological diseases^43–45^.

The behavioral indicators highlighted are also more asymmetric in performance between the two arms in MS subjects, reflecting the fact that MS is a pathology that can affect both sides of the body in an asymmetric way^46^. This was the case for 6 out of 11 subjects (S1, S2, S7, S8, S10 and S11) participating in our study.

The activity of shoulder muscles was different in MS subjects with respect to control subjects only in movement tasks. In particular, we observed a significantly different modulation and timing of the RHOM and DANT, when subjects performed movements toward their body. This result was consistent in the majority of the MS subjects. However, not much is known about the multiple factors influencing the abnormal posture in the upper body, including the site and severity of the neurologic damage. In our work, we observed that shoulder muscles might be modified both by the abnormal activation of a few muscles, as in the case of the RHOM and DANT, and, as discussed below, by a reorganization of muscle coordination.

Furthermore, despite the activity of many arm muscles there was a similarity between populations for many muscles, all MS subjects showed an evident biceps-triceps co-contraction when reaching distal targets that required the extension of the elbow. To our knowledge, these results have never been reported before, mainly due to a lack of studies in upper limb muscle activity in the MS population. Previous studies have focused on lower limb describing frequent functional impairments in balance^47^ and mobility, associated with spasticity^13^. Usually, extensor spasticity involves the quadriceps (muscles on the front of the upper leg) and the adductors (inner thigh muscles), and the trunk muscles in MS subjects with moderate impairment^48^.

The difference in modulation and activation of some muscles in MS subjects suggest to further investigate the organization of muscle activity. Synergy analysis has highlighted coordination deficits in other chronic neurological diseases, such as stroke^30,31^ and spinal cord injury (SCI)^49,50^. In SCI, the reorganization of muscle synergies seems always to occur in upper and lower extremities^49,50^, while, in some cases, it is also possible to observe the alteration of the number of synergies and activation profiles^51^. After a stroke, the preservation of the organization of muscle synergies depends on the level of impairment. A study^31^ involving subjects with a different range of impairment levels during reaching task, confirmed preservation of muscle synergies in mildly impaired stroke survivors, but reported evidence of synergy merging and fractionation in severe impaired subjects. Instead, the activation profiles of muscle synergies showed that the synergies dominated by the activation of shoulder muscles are generally altered both in chronic^43^ and acute^32^ stroke survivors with moderate impairment of the upper limbs. To our knowledge, this is the first study investigating muscle synergies in the upper limbs of subjects with MS. The results revealed no difference in the number of synergies between control and MS subjects. However, our MS population was characterized by mild to moderate upper limb impairment and it is possible that a change in number of synergies might be observed only with a higher level of impairment in the upper extremity. In fact, some studies^31,52^ suggested that the dimensionality of muscle synergies is correlated to the level of the neurological motor and functional impairments. Further investigation of more severely and diversely impaired MS subjects is necessary to support this hypothesis more conclusively. The number of muscle synergies both in the movement and isometric tasks was consistent with what has been already found by others with similar tasks^26,30,43^. The isometric task was characterized by a smaller number of synergies than the movement tasks for both populations. This was expected since in this case the movement was absent^53^.

For both populations, the analysis identified three primary synergies which involved the distal muscles, another synergy that involved proximal muscles and the last synergy included shoulder muscles, as also observed by other authors^26,54^.

We also investigated the effects of different environments on muscles coordination patterns as described by muscle synergies. In free space (FS) and when an unusual resistive (RF) or assistive (AF) force was added to the environment, the subjects with MS modulated their synergies in ways that differed from the control subjects. During movement tasks, the MS subjects changed to a greater extent the organization and the activation profiles of their muscle synergies in the presence of external forces. MS might influence the ability to generalize and adapt muscle activation patterns to different movement conditions, i.e. to the presence of forces. Thus, specific modifications of the structure of the muscle synergies might reflect a strategy adopted by the impaired subjects to perform movements in response to changes in the environment, or it might be just an adverse consequence of the disease.

We found that the structure of muscle synergies (weight) between D and ND arm was preserved in all different tasks, indicating that the same modular structure is adopted by both populations to perform reaching with the D and ND hands.

The asymmetric performance of movements in subjects with MS seems mainly due to an increase of the asymmetric activation of muscle synergies between sides. Indeed, we observed that the activation of upper limb muscle synergies was significantly different between arms in controls and MS subjects. In particular, the activations of muscle synergies in the MS population was more dissimilar between sides than the one in controls.

Since the environmental conditions were the same and the influence of handedness was similar in the two populations, this asymmetric activation of muscle synergies between sides in MS subjects might be due to the pathology.

Our study suggests that, duration, speed and accuracy of the reaching movements for the motor performance, and the activity of shoulder muscles as well as the muscle synergies (in particular, their organization and activation) for the muscle activation, might represent biomarkers, i.e. features that allow discriminating MS subjects with mild to moderate upper limb impairment from controls. The existence of these biomarkers opens the possibility to fully characterize upper limb motor impairment both at the behavioral and muscular level in people with MS, and to promote the development of personalized interventions for this population. Indeed, these results have important implications for the upper limb assessment and training of MS subjects, as they could provide guidance to promote functional recovery and to counteract the progression of the disability.

Concerning the functional evaluation, executing upper limb movements without external forces is a suitable and simple task to highlight meaningful functional impairments in MS subjects. However, these differences are more evident in the isometric task or when, in the movement tasks, the external forces change. The behavioral parameters - i.e. movement duration, average speed, smoothness and accuracy - were sensitive to alterations in movement execution due to the MS disease. Muscle amplitude and muscle synergy analysis revealed modifications of muscle activity in the MS population. In particular, muscle synergy analysis detected aspects related to the muscle coordination that were not evident from the analysis of single muscle activity. Consequently, the analysis proposed in this work appears to be a valuable tool to investigate motor impairments in people with MS. This protocol is easy to apply and well tolerated by the subjects, as indicated by the successful engagement of all participants to this study.

Concerning the treatment of upper limb motor impairments in MS, our results suggest that despite muscle coordination being affected, muscle synergies can be modified changing the environmental conditions. In rehabilitation, it is still an open question if complete recovery of motor abilities after neurological diseases may occur by resuming healthy muscle coordination or through the adoption of new solutions. Therefore, there is not yet a clear indication of which tasks and treatments are the best suited to optimize each individual’s motor recovery. However, our results add insights about the effect of different forces on muscle coordination of MS subjects with mild to moderate upper limb impairment: this is an important indication for the design of the specific training protocols once the rehabilitation goals have been established.

### Cautionary notes

This study is a proof of concept, based on 11 MS subjects with mild to moderate upper limb impairment. It is essential to enlarge the sample size of the MS population and to investigate subjects with different levels of upper limb impairment to further validate the results and have widespread indications for the design of assessment and rehabilitation protocols. In subjects with more severe impairment, these analyses could lead to different outcomes, as mentioned in the discussion related to the muscle synergies.

We could have performed the analysis on the more affected relative to the less affected arm. Such an approach is prevalent for the stroke population. However, in MS there is not a clear distinction between the affected and not affected arm as in stroke survivors, where this aspect is defined by location of the brain lesion. In MS there could not be a clear relation between the asymmetries in the brain and in the behavior. Moreover, MS affects people in different ways both at the neural and at the behavioral level, thus one or both sides can be affected, and they can be affected in a similar or a different way. Our subject population reflected this: about half of our MS population was strongly asymmetric for upper limb while the other was not. For these reasons, we preferred not to make any a priori (based on 9-HPT clinical test) assumption for the evaluation of the behavioral and muscle activity of the two arms. Moreover, all the subjects had a mild to moderate upper limb impairment and this impairment did not affect their handedness (this is not the case for severe upper limb impairment or in stroke survivors where the less affected limb often becomes the dominant one). Thus, in this population, handedness might impact motor performance similarly to healthy subjects, and maybe as much as their impairment, so we preferred to group dominant and non-dominant side. However, in a population more homogeneous with respect to asymmetry between the impairment of the two body sides or with higher impairment, an analysis comparing more and less affected sides might give clearer information on motor control issues in the upper arm of persons with multiple sclerosis.

We acknowledge that we adopted a self-selected speed rather than a fixed speed in order to favor the execution of the most natural and unconstrained reaching movements. Reaching speed has an impact on muscle synergies^26,27^, therefore we cannot exclude that it might have influenced our results.

The measure of maximal voluntary contraction would have simplified muscle activity and synergies analysis. We could not measure it because MS subjects are sensitive to muscle fatigue that would have compromised the measure itself and prevented participants from performing the proposed tasks.

## Materials and Methods

### Subjects

Eleven subjects with clinically definite multiple sclerosis (MS: 9 Female; 50 ± 10 years) according to McDonald criteria^55^ and eleven age and gender matched controls (C: 9 Female; 50 ± 9 years) participated in this study. The groups were equivalent in terms of hand dominance, age (F(1,20) = 0.21, p = 0.64) and gender. The inclusion criteria for MS subjects were: stable phase of the disease, Expanded Disability Status Scale (EDSS)^10^ ≤7, Fatigue Severity Scale (FSS)^56^ >=20, 9-Hole Peg Test (9-HPT)^57^ >20 seconds, and absence of treatment with corticosteroids within the previous three months. Notice that upper limb impairment was tested bilaterally by using the 9-HPT.

The control subjects had normal range of motion and muscle strength and no history or evidence of neurological or musculoskeletal disorders.

Subjects were tested for hand dominance based on the Edinburgh Handedness Inventor^58^. All MS and control subjects were right-handed with no problems of visual integrity, i.e. they could clearly see the information – target and cursor positions – that was displayed in the computer screen. Demographic and clinical data for each MS subject are listed in Table 1.

The study was approved by the local Ethical Committee (Comitato Etico Regionale Liguria, 06-10-2014, 201REG2014) and conformed to the ethical standards of the 1964 Declaration of Helsinki. Each subject provided written informed consent to participate in the study and to publish individual data.

### Set-up and Protocol

This study aimed at characterizing upper limb behavioral parameter, muscle activity and muscle synergies in the D and ND side while MS and control subjects performed reaching tasks in four different mechanical environments (Fig. 1B):

1. Free space (FS): the hand of the subjects moved unimpeded by external forces;
2. Assistive force field (AF): a constant assistive force field attracted the hand of the subjects toward the peripheral target (force amplitude: 5 N);
3. Resistive force field (RF): a resistive force field attracted the hand of the subjects toward the center of the workspace, i.e. an elastic force opposed the subjects’ movements toward the peripheral targets (linear spring stiffness coefficient was 15 N/m);
4. Rigid constraint (isometric force task, IF): the subject’s hand applied isometric forces to reach the peripheral targets.

In the isometric task (IF) subjects held a fixed force sensor (Gamma SI 13010, ATI Industrial Automation Inc.). In the movement tasks (i.e. FS, AF, RF), subjects grasped the handle of a planar manipulandum^59^; Fig. 1A. The robot recorded the end-effector position and provided the external forces.

Subjects were seated on a chair and the position of the seat was adjusted to keep the arm approximately horizontal at shoulder level; the movements were restricted to the horizontal plane, with no influence of gravity. A 19′ LCD computer screen was placed vertically in front of the subjects, about 1 m away, at eyes level.

The subjects reached targets positioned in eight directions equally spaced from a central target, i.e. at 14 cm distance on the screen. This distance corresponded to a displacement of 14 cm of the end-effector in the FS, AF and RF tasks and to a 10 N force step for the IF task; Fig. 1B. Each target (green circle, 10 mm radius) was presented five times in random order. Therefore, subjects performed 40 center-out movements per task, i.e. a total of (40*4 tasks) 160 center-out movements for each arm. Each target was presented again only after all eight targets had been reached. The cursor (yellow circle, 5 mm radius) position correspondent to the motion or the force applied at the robot end-effector was continuously displayed during the execution of all tasks. All subjects started the experiment with the dominant (right) arm. The different tasks were presented in random order within each arm. Subjects were asked to reach the targets as accurate as possible, without time constraints, thus they perform the task at their self-selected speed.

Muscle activity was recorded with surface electrodes for electromyography (CometaWavePlus, Cometa Srl, Italy). Electrodes were placed according to guidelines of the Surface Electromyography for the Non-Invasive Assessment of Muscles European Community project – SENIAM^60^ and Anatomical guideline^61^. The activities of the following 15 muscles were recorded from each upper limb: triceps brachii long (TRLO) and lateral head (TRLA), biceps brachii short (BICS) and long head (BICL), brachioradialis (BRAD), brachialis (BRA), pronator teres (PRON), infraspinatus (INFR), latissimus dorsi (LATI), upper trapezius (TRAP), rhomboid major (RHOM), pectoralis major (PECT), anterior (DANT), medial (DMED) and posterior (DPOS) deltoid. The protocol required a minimum of two minutes break between each task. Subjects were allowed resting when and as long as they needed; the experimental sessions lasted about two hours.

### Data Analysis

#### Behavioral parameters

Movement and force trajectories were sampled at 60 Hz and smoothed by using a sixth order Savitzky– Golay filter (cut-off frequency: ~11 Hz for the movement signals and ~8 Hz for the force signals), which was also used to estimate the subsequent time derivatives of the trajectory. We focused the analysis on the center-out cursor movements. The movement onset was defined as the first time instant the cursor speed exceeded the threshold of the 10% of maximum peak speed^41^. The movement ended when the cursor was inside the target and the speed underwent and remained under the same threshold^41^.

We analyzed the following performance indicators:

- Movement duration (s): time elapsed between the onset and the end of the cursor movement^15^.
- Average speed (m/s): average speed of reaching of the cursor movement.
- Normalized Jerk index (adimensional): the square root of the jerk; i.e. the third time derivative of the cursor movement trajectory in the movement tasks, averaged over the entire movement duration and normalized with respect to duration and path length^62^.
- 100-ms aiming error (deg): the angular difference between the target direction and the actual movement direction, estimated in the first 100 ms of the movement^7^.
- End-point error (m): the distance between target position and cursor position when the speed felt below 10% of the maximum speed for the first time^63^.
- Linearity error (%): the percentage increase in the length of the trajectory traced by the cursor with respect to the nominal trajectory, i.e. the straight line that connects the initial and the final points of the trajectory^7^.
- Lateral deviation (m): the maximum lateral distance between the cursor trajectory and the nominal trajectory^15^.

These indicators were computed for the dominant and the non-dominant arm and they were compared to highlight differences due to handedness.

However, since MS could affect either the dominant or the non-dominant side, when we average these indicators across the entire MS population we might lose the information about asymmetries between body sides due to the pathology. Therefore, we investigated the similarity between the behavioral indicators of the two arms by computing also the absolute difference between the indicators of the two arms for each subject in all tasks.

#### EMG pre-processing

EMG signals were acquired at 2 kHz; band-pass filtered (30–550 Hz), rectified, low-pass filtered (cutoff: 10 Hz) to obtain the EMG envelopes^30,31^. To correct the inter-arm EMG-amplitude differences due to electrode placement and to ensure that the extraction of the synergies would not be biased against the low-amplitude muscles, the envelope of each muscle signal was normalized by its median value obtained over all tasks. The normalization based on the median value instead of the maximum is more robust to outliers^30^.

#### Muscle activation

The normalized EMG envelope for each subject, arm, task and repetition was segmented in the 8 directions. For each trial, we considered a time window starting 250 ms before the movement onset^64^ and ending with the movement. We computed the root mean square values (RMS) and we averaged them across the five repetitions for each target direction. Then the normalized EMG envelope of each muscle related to each repetition, direction, task, arm and subject was resampled on 100 time points. We calculated the Pearson correlation coefficient (r) to compare the difference in the modulation of EMG data (i.e. waveforms)^32,40,65,66^ between D and ND arms and among tasks (r_EMG-ARM_, r_EMG-TASK_, respectively).

#### Muscle synergies

In recent years, several mathematical techniques have been developed to facilitate the analysis of complex muscle activation patterns. Matrix factorization techniques attempt to model complex multivariate data as linear combinations of a small set of basis vectors. Their application to EMG data provided evidences that the normal motor control may be based on the use of a relatively limited set of muscle synergies, each representing a muscle activation pattern with a specific organization and temporal profile^36,65,67,68^.

For each subject, arm and task, we extracted muscle synergies from a matrix including the resampled normalized EMG envelopes related to the eight directions averaged across repetitions, by using the non-negative matrix factorization algorithm^39^. The NNMF algorithm decomposes the EMG envelope in a defined number of positive components or muscle synergies. The organization of a synergy is determined by the contribution (i.e. weight coefficient) of each muscle, as specified by the weight matrix W. Its activation profile is defined by the activation coefficients, specified by the matrix H^39^. Since, the iterative algorithm can find a solution as a local and not global minimum, the extraction was repeated fifty times and the solution explaining the highest overall amount of variance was selected^30,31,37,69^. The algorithm requires as input the number of muscle synergies to extract. Synergy extraction was repeated from one to the number of recorded muscles, thus in our case we obtained 15 sets of muscle synergies for each subject in each task. For the subsequent analysis, we retained the minimum number allowing a good representation of the original muscle activation.

For each subject, to objectively determine the minimum number of muscle synergies required to reconstruct each data set, we used the higher number obtained from two different methods based on the inspection of the R^2^ curve that represents the fraction of total variation explained by the synergy model^26^.

The first method estimated the minimum number of synergies that achieved a R^2^ > 90%^26,70^. The second method was based on the detection of a change in slope of the R^2^ curve^70^. A series of linear regressions were performed on the portions of the curve included between the N-synergy (N = 1 to 15) and its last point (i.e. 15^th^ synergy). N was then selected as the minimum value for which the mean squared error of the linear regression was less than 10^−4^. In case of mismatch between the two criteria, the larger N was chosen^70^.

In order to simplify the analysis, the same number of muscle synergies, corresponding to the rounded average across subjects, was retained in the same group and arm.

The order of muscle synergies as output of the NNMF might differ among tasks, arms and groups. Therefore, we ordered them looking at the similarity of the W, in terms of maximum scalar product between the W of each pair of synergies^71^.

Since we observed that the number of muscle synergies was equal between arms and subject groups, we created a set of reference synergies for each task. To do that, first we pooled in a unique matrix the W related to D and ND arm of all control subjects. Subsequently, we computed on the matrix a hierarchical clustering procedure based on the minimization of the Minkowski distance in according to Cheung *et al*.^30^. The number of clusters was equal to the number of muscle synergies extracted for each task. We obtained the set of reference muscle synergies by averaging the synergy vectors within each cluster. Then we ordered the synergy vectors for each subject, task and arm separately using the correspondent set of reference synergies.

To evaluate the similarity of the weight coefficients (i.e. W) between control and MS subjects we used the scalar products (DOT)^26,36^. More specifically, for each synergy the weight coefficients of each MS subject were compared with the weight coefficients of the control subjects; then we calculated the mean values across muscle synergies and then across individuals (DOT_INTER-GROUPS_). This procedure was implemented for each arm and task.

The scalar product was used also to estimate the similarity of the weight coefficients of the muscle synergies between D and ND arm (DOT_ARM_) within each group for each task, and to estimate the similarity across tasks (DOT_TASK_, i.e. FS vs AF, FS vs RF and FS vs IF) within each group and arm.

We evaluated the similarity of the activation of muscle synergies (i.e. H) by using the Pearson correlation function^32,40^. Analogously to the W, we estimated the similarity between groups (r_INTER-GROUP_), and between arms (r_ARM_) and among tasks (r_TASK_, i.e. FS vs AF, FS vs RF, FS vs IF).

To obtain a reference value to assess the degree of similarity between groups the weight and the activation coefficients of each control subject were compared with the weight coefficients of all other controls and then averaged across muscle synergies and across individuals (DOT_INTRA-GROUP,_ r_INTRA-GROUP,_ respectively)^40^.

### Statistical analysis

To test if the indicators related to behavioral performance, the number and the similarity measures of muscle synergies differed between the two subject groups and depend on the task or on the arm dominance, we ran repeated-measures analyses of variance (rANOVA) with two within-subjects’ factors: “task” (1–4: FS, AF, RF and IF), “arm” (1–2: D and ND arm); and one between groups factor, “disease” (1–2: C and MS). Furthermore, for the muscle activation patterns (RMS), to investigate specific differences in movements that required arm flexion or extension we added another within-subjects’ factor, the “target direction” in the repeated-measures ANOVA. Finally, to investigate if the indicators of similarity between the two body sides in terms of behavioral indicators and muscle synergies (i.e. DOT_ARM_ and r_ARM_) differed between the two subject groups and depend on the task, we ran a rANOVA with one within-subjects’ factors: “task” (1–4: FS, AF, RF and IF), and one between groups factor, “disease” (1–2: C and MS). The assumption of sphericity was tested on each variable using Mauchly’s test. If the assumption was rejected the Greenhouse-Geisser correction was applied. Post-hoc analysis (Fisher’s LSD test) was used to verify statistically significant differences obtained with repeated measures ANOVA. The significance level was set at p < 0.05. The statistical analysis was performed within Statsoft environment (Statistica software 7.1).

## Electronic supplementary material

Supplementary information
