# Supplementary material for: Evaluating upper limb impairments in multiple sclerosis by exposure to different mechanical environments
Source: Sci Rep. 2018 Feb 1;8:2110. doi: 10.1038/s41598-018-20343-y (PMC5794735; doi:10.1038/s41598-018-20343-y)
Supplement: Supplementary file 1 — Supplementary information [file 41598_2018_20343_MOESM1_ESM.pdf]

# Evaluating upper limb impairments in multiple sclerosis by exposure to different mechanical environments

**Laura Pellegrino[1,*], Martina Coscia[2,3], Margit Muller[1], Claudio Solaro[4], and Maura Casadio[1]**

[1]*Dept. Informatics, Bioengineering, Robotics and Systems Engineering, University of Genoa, Italy*

[2] *Bertarelli Foundation Chair in Translational Neuroengineering, Ecole Polytechnique Federale de Lausanne, Lausanne, Switzerland*

[3] *Wyss Center for Bio- and Neuroengineering, Geneva, Switzerland*

[4] *Azienda Sanitaria Locale di Genova ASL3, Genoa, Italy*

*** Corresponding author:**
**Laura Pellegrino**
**Dept. Informatics, Bioengineering, Robotics and Systems Engineering, University of Genoa**
**Via Opera Pia 13**
**16145 Genoa, ITALY**
**Phone: +39 010 3536550**
**E-mail: laura.pellegrino@edu.unige.it**

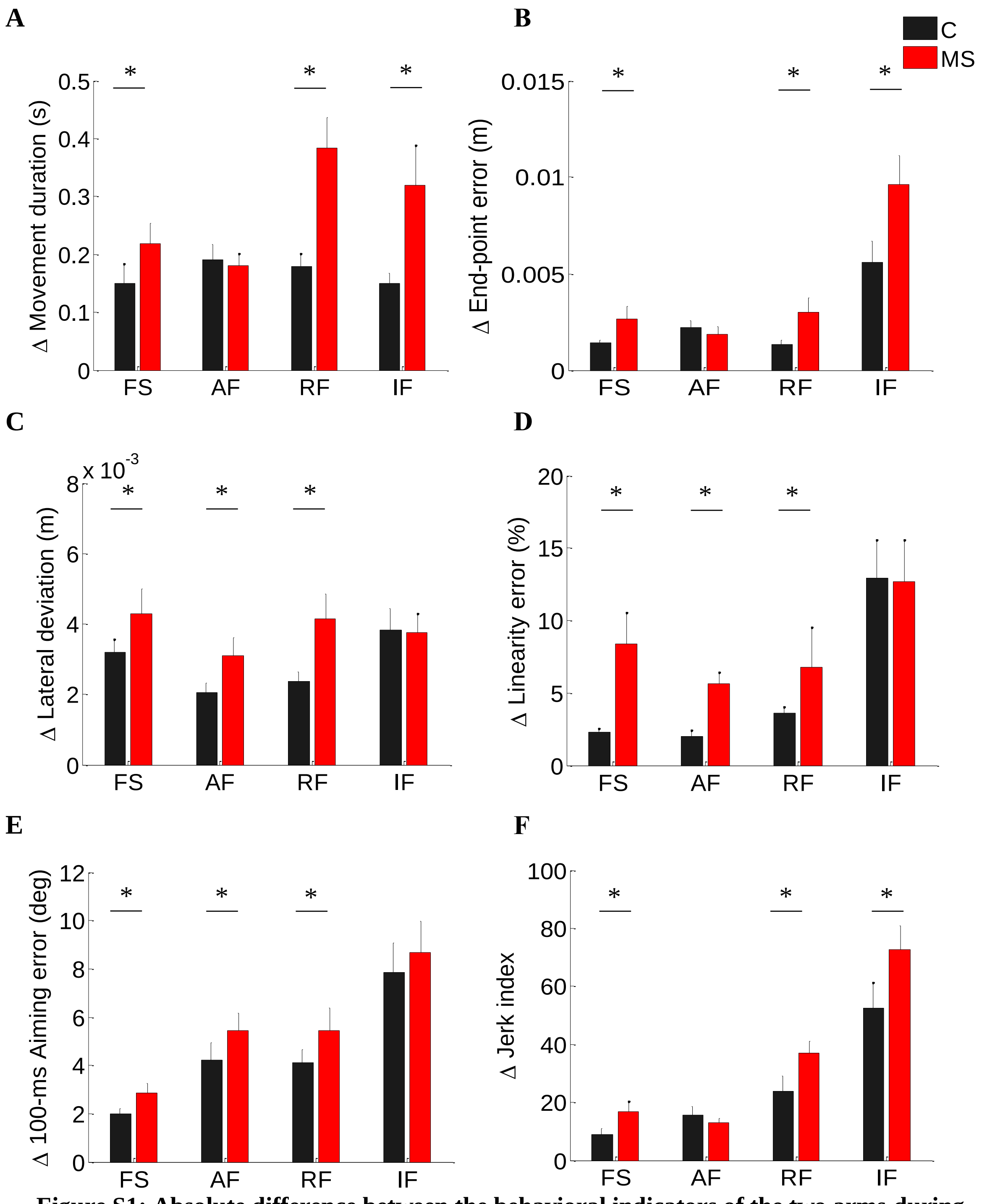


**Figure S1: Absolute difference between the behavioral indicators of the two arms during the motion tasks (motion trajectories) in absence of external force (FS), in presence of assistive (AF) or resistive (RF) force and during the isometric task (IF, force profiles).** Control subjects (C) and MS subjects (MS) are shown with different colors as indicated in the legend. The error bars indicate the standard error of the indicators. * indicates significant differences ($p<0.05$) between subject groups (C vs MS) for each task.

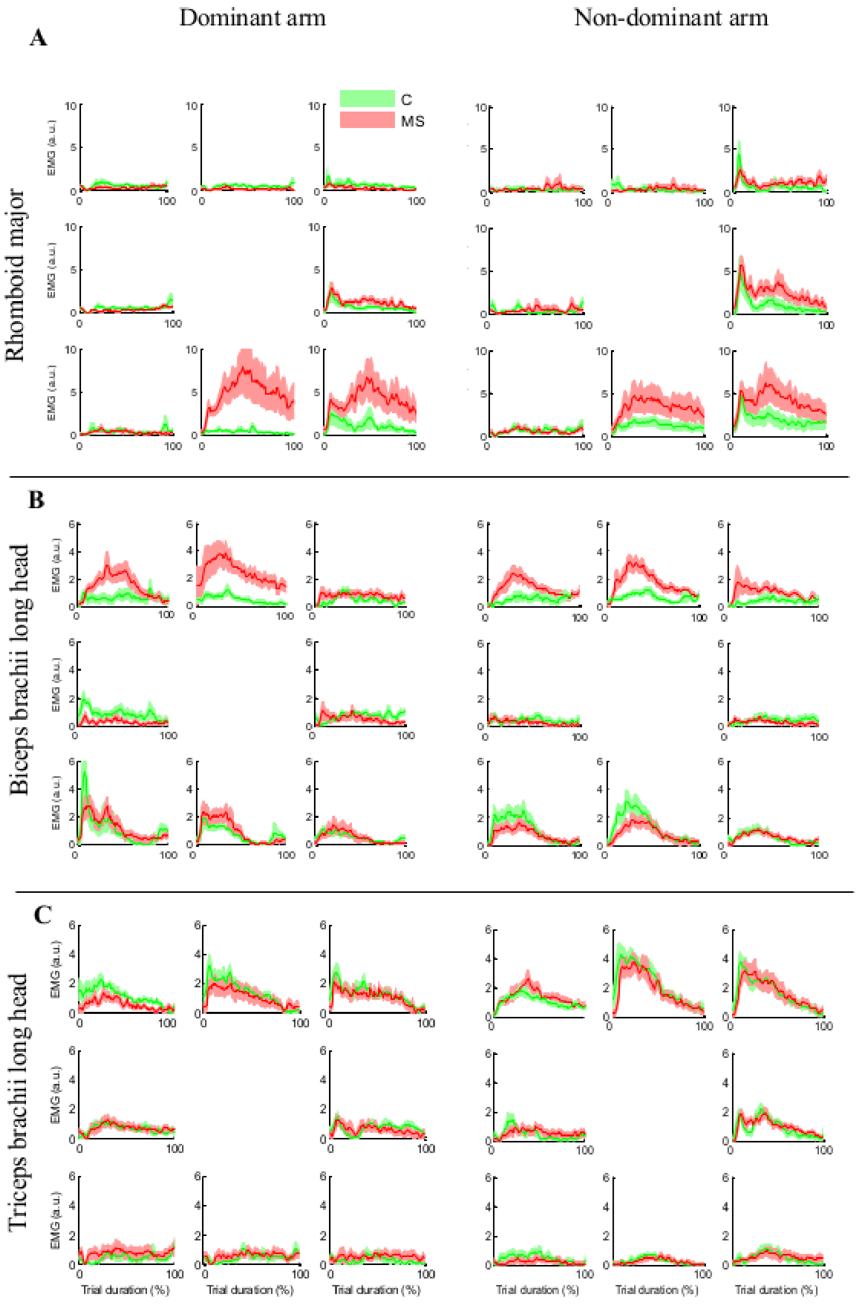


**Figure S2: Normalized EMG envelope time series.** Activation of the rhomboid major (RHOM, panel A), biceps brachii long head (BICL, panel B) and triceps brachii long head (TRLO, panel C) during reaching movements in the eight directions in the free space task. The green and red line respectively represents the mean for control subjects (C) and multiple sclerosis subjects (MS). First column: dominant arm; second column: non-dominant arm. The shaded area indicates the standard error.
